# Better together: a community- hospital integrative model of healthcare as a practical solution for providing excellence in endocrinology care in an era of limited resources

**DOI:** 10.1186/s13584-015-0024-9

**Published:** 2015-07-01

**Authors:** Anat Jaffe, Aviva Yoselis, Liana Tripto-Shkolnik

**Affiliations:** Endocrinology and Diabetes Unit Hillel Yaffe Medical Center Hadera, Hadera, Israel; Viva Research Institute, Jerusalem, Israel; Endocrine and Diabetes Service, Meuhedet Health Fund, Ra’anana, Israel

**Keywords:** Continuity of care, Diabetes, Endocrinology, Integrative Model of Healthcare

## Abstract

**Background:**

The demand for endocrinology services is growing worldwide, particularly among minority and underserved populations, mainly due to the rapid global increase of diabetes. The medical education of endocrinologists is a resource consuming process and is mainly hospital-based. Yet, given the chronic nature of endocrine morbidity, the greatest demand for endocrinology services is in the community. However, an isolated endocrinologist cannot cope with the rapid changes in the field. Limited funding of hospital facilities does not allow for the establishment of a freestanding endocrine-center; thus, the Community- Hospital Integrative Model of Healthcare (Co-HIMH) was developed and implemented in an Israeli government hospital and is presented as an approach for achieving excellence in endocrinology care.

**Aim:**

To describe the design, function and challenges of the Co-HIMH.

**Model description:**

Originally, three pillars: 1) the hospital unit as a regional expertise resource, 2) Co-HIMH endocrine providers participating in both community and hospital services, and 3) integrated information flow between health-care providers, supported the integration between hospital and community networks.

**Results:**

The community and hospital endocrine human resources were increased to create attainable and accessible endocrine services in the community and hospital. Collaborative interaction between healthcare providers increased both continuity of care and efficient patient navigation. Endocrine hospital referrals for specialized procedures have grown. Within this area of low socioeconomic status, continued medical endocrine education was conducted introducing state-of-the-art treatments. The essence of these achievements was maintained by continuous training of fellows. During the years that the Co-HIMH operated, it certified 14 % of all endocrinology fellows in Israel. Unresolved issues regarding employee rights and formalization of the Co-HIMH status are significant challenges.

**Conclusions:**

In the era of limited resources and increased healthcare demand, creative infrastructures are required. This article provides a successful example of a preliminary model and proposes future needed modifications.

## Background

**Fig. 1:**
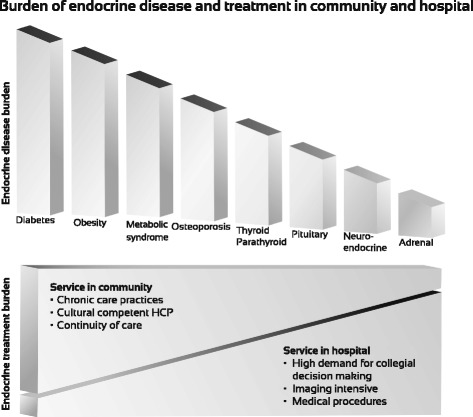
Endocrine disease and treatment burden in community and hospital. The division between disease burden and treatment are displayed. Community-Hospital Integrative Model of Healthcare offered practical means for an efficient way to implement this dogma

The demand for endocrinology services is growing worldwide, mainly due to the rapid increase of diabetes, obesity, the metabolic syndrome and osteoporosis and is particularly notable among minority, immigrant and socioeconomically disadvantaged populations [1–5]. Chronic endocrine diseases may cause serious complications, including disability and death, imposing major social and economic costs on patients, their families and society at large [6–8]. Some other classic endocrine diseases (i.e. thyroid, adrenal, pituitary, and neuroendocrine pathology) are of lower incidence and are thus a challenge for diagnosis and proper management. This heavy and diverse burden of endocrine disease requires efficient handling of the treatment by primary and secondary physicians in the community, and, in special circumstances, the hospital, setting (Fig. 1). To accomplish this, an ongoing adaptation of the healthcare system is required. Although the greatest demand for endocrinology services is in the community, endocrinology fellowships, continuing medical endocrine education (CME) and research, collaborative consults, exposures to rare endocrine diseases, and access to acute care patient services, are almost always hospital-based. Further, with the rapid change in the perception of current endocrinology towards patient-centered individualized treatment plans, including thyroid and neuro-endocrine oncology, the scene of a single physician managing the patient’s ‘case’ in an isolated clinic is becoming rapidly obsolete. Against this background, the paucity of hospital based endocrine procedures coupled with system resource shortages led healthcare policy makers to question the need for hospital-based endocrinology. However, policy makers are often not aware of, or perhaps do not take into account, the fundamental role of intra-hospital collaboration and hospital-based CME and research designed to ensure treatment expertise and to ultimately save lives and potentially reduce future costs.

The endocrinology profession is currently at a crossroads, facing the challenge of how to provide healthcare services that will combine hospital-based expertise while addressing community needs. This article presents an operative system model that was designed to adapt the changing endocrinology field by effectively utilizing hospital and health fund resources.

In this paper we describe the rationale, development and implementation of the Community- Hospital Integrative Model of Healthcare (Co-HIMH) as it was applied in a peripheral government hospital in Israel. We also note difficulties encountered and future challenges in implementation.

### The Israeli healthcare system

Every resident of Israel is entitled to basic health care as a fundamental right under the National Health Insurance Law. The law declared a system of public funding to the health funds according to a capitation formula based on number and age of members in the fund. It also determined a uniform “benefits package”, a list of medical services and treatments that each of the four competing health funds is required to provide to its members. Every resident has a right to register as a member of a health fund of his/her choice, free of any restrictions or limitations. The health funds provide primary care services; one fund provides direct hospital care in certain geographic areas. The government regulates prices and policy, but also provides some health services as the main proprietor of Israeli hospitals.

## Methods

### The Community- Hospital Integrative Model of Healthcare (Co-HIMH) in endocrinology

Our model draws on the Chronic Care Model (CCM) [9–11], Patient Centered Care Model (PCC) [12] Cultural Competence, [13], and Continuity of Care concepts [14, 15]. Each of these models addresses specific needs of chronically ill patients living as members of a culturally diverse population, who encounter financial, cultural, linguistic and service oriented barriers to care. These models have been recognized both internationally and within Israel as important milestones within the healthcare system to elevate the quality of care and reduce patient morbidity [16–18].

However, the integration of endocrine services between the community and hospital settings, which include both supervision of patient navigation and collegial collaboration within these complementary medical systems, is not sufficiently addressed in the aforementioned models.

The Co-HIMH rests on three essential theoretical pillars:

1. The Endocrine unit in the hospital acts as a regional expertise resource (including education and research) for community health care providers (HCPs), hospital HCPs, patients and the community at large.
2. The majority of endocrine services are provided within community clinics, but all endocrine staff participates in hospital endocrine services.
3. Integrated informational flow is mediated by both efficient digital data transfer (medical data) and interpersonal meetings at the community and hospital, supplying patient medical, behavioral, and complementary health information.

#### Goals of the Co-HIMH

The needs, rationale and the model pillars were translated into specific goals:

1. *Create a regional knowledge resource center*
2. *Improve continuity of care and professional supervision between community and hospital*
3. *Enable increased cultural competence in treating the diverse populations within the region*
4. *Increase the level of chronic illness prevention within the community*
5. *Implement the principles of chronic patient care within both the hospital and community*
6. *Reduce the number of emergency hospitalizations*
7. *Reduce overall costs of the chronic illness healthcare burden*
8. *Expand and strengthen the endocrinology profession in Israel*

### Development and implementation of the Co-HIMH

The Co-HIMH was developed and implemented in the Hillel Yaffe Medical Center (HYMC) during the years 2001–2013. HYMC is a government-owned hospital situated halfway between two major cities, Tel-Aviv and Haifa. It serves a population of ~ 450,000 individuals, many of whom come from lower socioeconomic communities (44 % with Socio Economic Rank (SER) ≤ 4 out of 10) [19]. The population is composed of heterogeneous communities living in urban, rural, village, or kibbutz communities, with Jewish, Arab and new immigrant residents, many of whom are from Ethiopia and the Former Soviet Union. Health services, especially in the sub-specialties, are limited; patient’s choice of provider and mobility among treatment plans are difficult [20, 21].

Until 1997, there was no established endocrine facility within HYMC and very few endocrinologists were available in the community based clinics. The endocrine service then received funding for only one institutional position, that of unit manager. The unit established cooperation with all four Israeli health funds, some with complete partnership and others with limited interaction. The “complete partnership” relationship between the unit and the health fund created a professional interaction that allowed for continuity of care, collaboration and the evolvement of the Co-HIMH. The health fund continuously funded one or two full-time positions for fellowships in endocrinology. Employment was provided by the research fund within the HYMC. (A research fund is a formal organization which has the legal right to utilize and procure health services within an Israeli hospital as well as employ staff). In return, the physicians were committed to providing 50 % of weekly hours per position and the rest of the hours in HYMC. Upon graduation, five expert endocrinologists continued in full-time positions within this infrastructure.

Therefore, the majority of endocrine services provided in the community of the partner health fund was by endocrinologists working in the unit or previous graduates of the unit who kept in contact, collaborated, and participated in weekly team meetings, all supported by the partner health fund.

## Results

### Creating a regional knowledge center

The unit continually trained residents to offer them accreditation in endocrinology (a process of approximately 2.5 years). As a result, knowledge and new medical developments continued to evolve for the entire Co-HIMH team. The size of the unit’s human resources allowed for the development of sub-specialties such as diabetes, osteoporosis, thyroid, and endocrine hypertension. These specialties enriched the knowledge base of the participating staff as well as improved the quality of care. The placement of the endocrine unit within the hospital allowed for open communication with physicians from other specialties such as radiology, nuclear medicine, pathology, and surgery. Their contribution was reflected both in the diagnosis and course of treatment. In this way, physicians from varied specialties collaborated and shared information about various types of illnesses and rare endocrine diseases. The unit staff taught endocrinology to residents from other specialties, internists, family medicine, and gynecology, as well as nurses and nursing students. Knowledge acquired within the unit was also transmitted to the community through dialogue and consultation regarding shared patients and CME for family physicians and trainings for nursing staff and patients. In this way, the unit became a regional knowledge center that both contributed and received informational resources.

### Improving continuity of care and professional supervision between community and hospital

#### Continuity of care

All of the unit physicians worked in community clinics as well as in the hospital endocrine- unit. This enabled them to see patients during morning regular clinic hours, and not only in the evening, as often happens with community endocrinologists’ appointments. This presence during the regular clinic work day allowed for more interaction and direct personal acquaintance with other physicians, as well as with allied health and administrative staff in the clinics. The ability of the hospital endocrinologists to directly access the health fund administrative system greatly enhanced the ability to prescribe necessary testing, issue prescriptions, refer for additional treatment and establish dates for further medical procedures. This access is reflected in the increase in the number of community clinic visits as seen in Table 1. In most systems, the hospital endocrinologist requests these services, but the patient must return to the community-based physician who actually prescribes the procedures. Co-HIMH prevented the patient from “getting lost” within the system, or not receiving the necessary referrals for further treatment because they were waiting for a clinic physician appointment.

**Table 1:** Co-HIMH -services [monthly average] and human resources allocation from 1998-2013

	1998	2001- Co-HIMH begins	2004	2007	2010	2013
Inpatient services	n/a	36	44	59	64	93
Outpatient clinic visits	46	87	71	236	177	231
FNA-U/S procedures	2	4	8	26	22	23
Ambulatory dynamic testing	7	8	12	10	13	5
Documented virtual visits^a^	0	0	0	n/a	n/a	57
Community clinic visits^b^	0	320	485	650	650	675
Patient Participation in Clinical Trials	0	35	7	6	18	29
Non-RCT [number]						
Clinical studies	0	2	0	1	2	7
Basic studies	0	1	1	2	0	0
Community based prevention programs	0	1-Tene-Briut located within the unit	1-Tene-Briut located within the unit	1-Tene-Briut as NGO 2- JAZ^2^	1- NGO 2- JAZ^2^	1-NGO 2- JAZ^2^ 3-Arab^3^
Human resources: Number of staff positions						
Hospital based^c^	0.75	1	1	1	1	1
Health fund based^d^	0	2	3	4	4	4
Total in hospital	0.75	1.6	2	2.3	2.3	2.3
Cumulative number of graduated fellows	0	0	1	4	7	11

^a^Includes digital, fax and telephone communication

^b^Based on extrapolated figures from the partner’s health fund data. Exact numbers are not available since several staff held positions with more than one health fund and this data was not accessible

^c^Full time position financed by the hospital

^d^Full time position financed by the health fund yet officially employed by the HYMC research fund; 50 % of the hours were dedicated to the community clinic, and the rest were divided between the hospital unit, ~33 %, and employee benefits [e.g. vacation, sick days, study days, maternity leave, and military reserve duty]

1-Health promotion organization for Israelis of Ethiopian origin, http://www.tene-briut.org.il

2-Endocrinology clinic in Jisr az-Zarqa together with Clalit Health Services and the NGO Bridge to the Future http://www.btf.co.il)

3-Diabetes prevention and treatment within the local Arab Israeli communities in conjunction with the Mehuedet Health fund. Abbreviations: FNA U/S- fine needle aspirations [or core biopsy] guided by ultrasound, NGO- Non-Governmental Organization,Non RCT- non Randomized Controlled Trials

#### Flexible supervision of patients between the hospital and community systems

Most of the visits of the partner health fund’s patients in the hospital were for the purpose of obtaining complicated diagnostic procedures (endocrine dynamic tests, CT scans, nuclear medicine, invasive procedures) and/or treatment (IV drug infusion, surgery). Many of the endocrine-based ER visits for urgent care or further inquiry were reduced to true emergencies. After the completion of short term and intensive treatment in the hospital, the patient was transferred to the community team, facilitating continuity of care and collaboration.

### Culturally congruent and patient centered care

Over the years, the continued recruitment of a large number of highly qualified physicians allowed for the development of a diverse medical team that represented the cultural and ethnic diversity of the area served by HYMC. The staff consisted of native born, immigrant, Jewish, Muslim, secular and religious physicians, and 50 % were women. The presence of such a mixture among the team led to the expansion and further understanding of various cultural patterns of thinking and response. It also enabled adequate linguistic and cultural congruity between patients and caregivers, furthering “medical accessibility”. In the community, being within the patient’s environment permitted an appreciation of the physical environment, customs, nutrition and culturally-based behaviors, thus helping the Co-HIMH team implement appropriate patient education programs. For example, one program explored foot washing prior to prayer for Muslims and its contribution to the incidence of foot mycosis.

### Increasing the level of chronic illness prevention within the community

The Co-HIMH enabled the creation and participation in the health funds’ community projects for health promotion and patient support, thus furthering the unit’s presence as a “regional knowledge center” (see item 1). Team meetings provided time to think outside the box and develop educational programs and community-based medical interventions in collaboration with key opinion leaders in the community

For example, a unique project to combat the increasing rate of diabetes within the Ethiopian community was created. This project grew and eventually became an independent organization “Tene Briut^1^” (Table 1).

### Implementing the principles of chronic patient care within both the hospital and community

The close relationship with the personnel in the community clinics improved the knowledge of both the Co-HIMH endocrinologists and community medical staff and facilitated interaction with chronically ill patients. Community nurses were empowered to help patients and their family members identify and address possible barriers that prevented the patients from fully implementing changes based on medical recommendations. Often, implementation of patient self-care skills began in the unit while the patient was hospitalized. Further training and support then occurred in the community, facilitated by collaboration between hospital and clinic nurses. Family physicians and allied health staff received guidance about special medical conditions that could upset the patient’s health balance (e.g. need to change the steroid dosage when fever develops in a patient with Addison’s disease).

### Reducing the number of emergency admissions

The volume of medical activity and income from clinical research (Table 1) helped build an extensive infrastructure that included, in addition to physicians, an administrative assistant, clinical research assistants, a nurse and a dietitian. This infrastructure not only improved ambulatory and hospitalized patient care but also allowed for patient communication through fax, telephone consultation and e-mails in the patient’s language as an alternative to actual in-person visits. Questions were addressed and frequently resolved regarding the urgent care in the event of adverse side effects from medication, endocrine imbalance or consultation regarding handling of drug therapy before medical procedure or religious fasting days. This continual access to information and consultation reduced patient anxiety and the incidence of both deteriorating medical conditions and the need for emergency intervention. This access to information is reflected in the number of virtual visits as seen in Table 1. In fact, this access intensified patient satisfaction and sense of security. In addition, the referrals to the HYMC for inpatient complicated procedures, including elective surgery (e.g. thyroidectomy, parathyroidectomy, bariatric surgery) grew significantly.

### Reducing costs

Collaboration with the health funds and resource pooling enabled the hospital to maintain an endocrine unit with skilled personnel, without additional costs and investments. This teamwork established a synergistic relationship and prevented duplication of services. Clinical effectiveness also increased, as the time to make the diagnosis and institute proper treatment were diminished and more costly and complex hospitalization procedures were reduced. *“The physicians of the Co-HIMH are placed as specialists within the community clinics, available to answer questions and resolve medical issues. Complicated cases that deserve in depth investigation or specific medical procedures are given priority while practitioners receive comprehensive guidance for needed testing, thus reducing the hospital work load and wasting valuable resources” (translated excerpt of a letter from a senior family practitioner submitted to the Minister of Health)*.

The presence of a sizable professional endocrine center allowed for the entry of pharmaceutical and medical device companies to utilize this resource for clinical trials, an essential source of sizable income for a medical center located in a an area of low socioeconomic status. Information regarding patient’s participation in those trials is seen in Table 1. The hospital unit research fund income from those trials allowed participation of the Co-HIMH physicians in international conferences, further expanding professional expertise and CME.

### Expanding and strengthening of the endocrinology profession in Israel

#### Within the community

The health funds received highly trained endocrinologists because their specialized training was ongoing. Co-HIMH endocrinologists and its graduates continued to work as a team upon return to the community clinics. Diagnoses and treatment were carried out under the guidance, support and supervision of the endocrine-unit team. Consultations regarding imaging tests and endocrine suppression/stimulation tests were possible. Multidisciplinary team discussions about cases were often held within the unit without requiring that the patient come to the hospital. This collaborative approach provided an alternative to a more fragmented care model that does not facilitate collegial communication and can lead to a decline in the endocrinologists’ professional skills [22].

#### Within the hospital

Hospitalized patients received attainable and accessible endocrine services as a result of the increased access to medical resources. They also benefited from the diverse professional capabilities of the team. *“The overall [patient] satisfaction is very high. Best marks were related to information delivery, communication with patients and the professional services offered by the staff” (translated excerpt from the executive summary of an evaluation study conducted by an external company solicited by the HYMC management).* Many ambulatory patients used the services of the endocrine unit at the hospital. Urgent care patients, whose medical conditions required treatment outside regular working hours of community endocrine clinics, and patients with multiple or complex morbidities were able to receive longer visits and additional treatments because of the multidisciplinary system and the expertise of sub-specialists in the unit.

The Co-HIMH generated a unique center with a high professional profile that became a magnet for physicians looking for a quality fellowship. After several years, it was possible to select the best, and most appropriate, candidates for the unit.

#### Nationally

During the years 2001–2013, 78 endocrine fellows graduated in Israel; 14 % of them were trained at the HYMC endocrine Co-HIMH (personal communication with the Scientific Council, the branch of the Israeli Medical Association that is responsible for the planning and supervision of the physician specialization system in Israel). Their training included the acquisition of tools to manage the treatment of diverse and disadvantaged populations. Moreover, this model allowed for the creation of a cohesive group of endocrinologists who were able to advocate for their patients. Specifically, the endocrinologists noted the unequal provision of services, medications and treatments in the periphery, as well as the need to find solutions to these problems. Examples of issues that were addressed by the Co-HIMH endocrine group were the lack of a formal and systematic updating mechanism to incorporate new blood tests into the Israeli ‘benefits package’. This lack creates disparities between the heavily urbanized central region of the country and the periphery, in funding vital blood tests in endocrinology and disruptions in the continuous supply of rare endocrine medications. *“The Co-HIMH provides professional, reliable, dedicated and continues endocrine services in an area containing many deprived communities which lack such services” (translated excerpt from a position letter written by all senior managers of Israeli endocrine institutions and units).*

### Discussion and limitations

As with all models, the interface between theory and reality creates a spectrum of challenges that must be addressed:

1. **Conflicts of interest** among hospital and health funds administrations. For example, providing endocrine services in the community decreased the number of endocrine out-patients visits; moreover providing emergent endocrine service at the hospital unit reduced the number of hospital admissions. Therefore, there was less economic incentive on the part of the hospital administration to encourage care that would reduce the income. Indeed, this loss of hospital income may have been one of the reasons why the program was not continued. Outside Israel, this conflict has been approached in several ways. In the United States, pilot projects within the Medicare system have created “Accountable Care Organizations”, health payment systems that are held accountable for both quality of care and cost reduction and are able to incentivize providers (either individuals or systems) to improve patient health and not reimburse procedures. One pilot system offers bonus payments to providers “*if their efforts to improve care through better care coordination and other delivery reforms translate into slower risk-adjusted health spending growth and improved performance on quality measures for the patients they serve.*” ([23], p.984) After three years, this program did show savings. However, this system depends on all providers being under one supervising body that both evaluates and rewards. Other studies that reviewed health organization efforts to reduce hospitalizations using care coordination and patient education, failed to reduce hospitalizations in 14 out of the 15 participating systems [24]. Further analysis determined that organizations who offered the following interventions were able to reduce hospitalizations: telephone calls instead of frequent meetings; occasional provider meetings; creating a communications center for providers; delivering evidence-based education to patients; providing effective medication management, and comprehensive post-hospitalization transitional care. Costs were reduced only if care management fees were modest and the provider enacted the interventions cost effectively [25].
2. De facto agreements between the Co-HIMH staff and the local health fund managers were not based on generalized agreements with the hospital or on specific health system policy. This situation created bureaucratic obstacles when referring patients to non-endocrine related services within the hospital. For example, the Co-HIMH physicians requested in-hospital performance of scans (CT, radio nucleic and U/S) to enable discussion with the imaging staff on suitable imaging protocols for complex cases and to diminish unnecessary duplication of imaging.
3. **Jointness** is an infrastructure adaptation needed for inter-organizational network functioning to achieve common goals by effectively using in-house resources and coordinated cooperation [26]. De facto jointness was the milieu that enabled the creation of the endocrine Co-HIMH since from the very beginning it was based on interpersonal agreements between key position holders (hospital administration, hospital endocrine unit and health funds). However, jointness is not an integral part of the Israeli civil society culture, thus the lack of the de jure agreements made the Co-HIMH vulnerable to changes in key position holders.
4. Governability on the part of the regulator (Ministry of Health) was limited. Thus, interests of the regional population, as well as national needs of the endocrinology profession, were not imposed on local players. These weaknesses of the Endocrine Co-HIMH in HYMC clarified the need to create a fourth pillar in the model, that of a formal managing body consisting of leaders within all the relevant institutions, including the regulator. The managing body would need to oversee and implement the following: a) goal setting in an adaptive and needs-oriented manner b) logistics c) performance monitoring and d) adaptation of ongoing administrative issues with hospital and health fund systems, mainly human resources, employee benefits, and labor relations.

### Conclusions

This paper presented the Endocrine Co-HIMH at HYMC that operated for more than a decade. It enabled ongoing improvement of professionalization, efficient supervision of patients between different treatment frameworks, participation in culturally appropriate prevention treatments at the community level, and improvement of the patient and provider’s personal experience. The professional success was based on the fact that it was located within the hospital, yet had a major place within the community. Endocrine services that are solely hospital-based, with no connection to the community, cannot optimally meet the needs of the chronically ill. On the other hand, endocrine services that are based solely in the community cannot cope with the provision of quality care and often result in a decline of the endocrinologists’ professional expertise. Additional research is needed to measure the impact of the model on patient morbidity, hospitalizations and mortality. Furthermore, an in depth analysis of this model is needed to modify it for implementation in other healthcare settings.

## Footnotes

1 http://www.tene-briut.org.il
